# Prediction of 30-Day Readmission After Stroke Using Machine Learning and Natural Language Processing

**DOI:** 10.3389/fneur.2021.649521

**Published:** 2021-07-13

**Authors:** Christina M. Lineback, Ravi Garg, Elissa Oh, Andrew M. Naidech, Jane L. Holl, Shyam Prabhakaran

**Affiliations:** ^1^Department of Neurology, Feinberg School of Medicine, Northwestern University, Chicago, IL, United States; ^2^Department of Neurology, Biological Sciences, Division and Center for Healthcare Delivery Science and Innovation, University of Chicago, Chicago, IL, United States; ^3^Department of Neurology, University of Chicago, Chicago, IL, United States

**Keywords:** stroke, readmission, machine learning, natural language processing, bioinformatics

## Abstract

**Background and Purpose:** This study aims to determine whether machine learning (ML) and natural language processing (NLP) from electronic health records (EHR) improve the prediction of 30-day readmission after stroke.

**Methods:** Among index stroke admissions between 2011 and 2016 at an academic medical center, we abstracted discrete data from the EHR on demographics, risk factors, medications, hospital complications, and discharge destination and unstructured textual data from clinician notes. Readmission was defined as any unplanned hospital admission within 30 days of discharge. We developed models to predict two separate outcomes, as follows: (1) 30-day all-cause readmission and (2) 30-day stroke readmission. We compared the performance of logistic regression with advanced ML algorithms. We used several NLP methods to generate additional features from unstructured textual reports. We evaluated the performance of prediction models using a five-fold validation and tested the best model in a held-out test dataset. Areas under the curve (AUCs) were used to compare discrimination of each model.

**Results:** In a held-out test dataset, advanced ML methods along with NLP features out performed logistic regression for all-cause readmission (AUC, 0.64 vs. 0.58; *p* < 0.001) and stroke readmission prediction (AUC, 0.62 vs. 0.52; *p* < 0.001).

**Conclusion:** NLP-enhanced machine learning models potentially advance our ability to predict readmission after stroke. However, further improvement is necessary before being implemented in clinical practice given the weak discrimination.

## Introduction

Nearly 800,000 patients experience a stroke each year in the USA (1). The cost of initial admissions for stroke averages US$20,000 while readmissions cost on average US$10,000 (1–3). Reduction in readmission is, thus, an important target to reduce healthcare costs and improve patient care. However, several studies have demonstrated that available prediction models for readmission perform modestly (4, 5). A better understanding of the causes leading to readmission and better prediction tools may allow hospital systems to better allocate resources to the patients who are most at risk for readmission (6, 7).

Prior efforts to stratify risk of readmission have utilized basic statistical models, such as logistic regression, with modest results (AUC range: 0.53–0.67) (5, 7, 8). However, these studies do not report results on a separate held out dataset thereby not addressing the generalizability of these results. Also, since these methods are trained and validated on the same datasets, the results are highly prone to be inflated due to overfitting. Furthermore, logistic regression base models are incapable of properly weighing the interactions between the complex variables in additive analyses (4, 9).

Machine learning (10) (ML) has emerged as a new statistical approach to overcome the limitation of non-linearity and improve predictive analysis in healthcare. Advanced ML methods have shown to be superior for predicting readmission in patients with heart failure (11). Furthermore, natural language processing (NLP) methods can be utilized to automatically extract much of the rich but difficult-to-access medical information that is often buried in unstructured text notes within electronic health records (EHR). There has been widespread interest to use ML in conjunction with NLP to build clinical tools for cohort construction, clinical trials, and clinical decision support (9, 12). There has been, however, no study to use NLP of clinical notes and ML to predict readmissions after stroke. We, therefore, sought to evaluate advanced ML algorithms that incorporate NLP features of textual data in the EHR to improve prediction of 30-day readmission after stroke. We also seek to evaluate our models on a separate held out dataset in order to test the generalizability of our results.

## Methods

### Cohort

Using the Northwestern Medicine Enterprise Data Warehouse (NM-EDW), a database that collects and integrates data from the EHR at Northwestern Medicine Healthcare (NMHC) system practice settings, we identified stroke patients hospitalized at Northwestern Memorial Hospital between January 1, 2011 and December 31, 2015. Inclusion criteria were age >18 years old. We defined stroke by ICD-9 codes 430–436 for hemorrhagic and ischemic stroke, excluding 432.x, and 433.x0, and 435.x for transient ischemic attack or asymptomatic cerebrovascular conditions. We excluded patients who expired during index hospitalization and those with psychiatric admissions due to privacy restrictions on access to this type of data in the EDW.

### Data Extraction

We obtained discrete structured variables and unstructured free-form text-based clinical notes from the EHR (Cerner, Kansas City, MO) pertaining to the index stroke hospitalization for all patients meeting study criteria from the EDW. The EDW currently contains clinical data on nearly 6.2 million patients dating back to the 1970s, which can be easily queried at the individual patient level or for aggregate data and can link laboratory tests, procedures, therapies, and clinical data with clinical outcomes at specific points in time.

For discrete variables, we recorded demographics (age, sex, race, ethnicity, insurance status, marriage status, smoking status), comorbidities based on ICD-9/10 codes (prior stroke, prior transient ischemic attack (TIA), hypertension, diabetes, coronary artery disease, hyper/dyslipidemia, atrial fibrillation, chronic obstructive pulmonary disease, hypothyroidism, dementia, end stage renal disease, cancer, valvular heart disease, congestive heart disease, prior coronary stent or bypass), prior healthcare utilization (number of ED visits and number of hospitalizations in the preceding year), stroke type (hemorrhagic vs. ischemic), length of stay, index hospital stay complications (pneumonia, mechanical ventilation, and percutaneous gastrostomy tube placement), discharge disposition, and discharge medications (e.g., anticoagulants). For non-discrete variables (e.g., text), a data analyst extracted the notes from the EDW. We included only a small appropriate subset of report types to identify potential predictors of readmission: admission, progress, consultation, and discharge notes. We pre-processed them to make it usable for machine learning and combined the raw text data with the discrete data, linking by a common identifier.

### Feature Selection

A feature is an individual measurable property or characteristic of a phenomenon being observed. We built different feature sets for our predictive models. First, we compiled discrete features, some of which were used previously in studies of readmission after stroke (Table 1). We then extracted these features from the structured data, when available, in the EDW. These 35 discrete features formed the first feature set. We ranked each feature based on its importance using feature importance methods. Specifically, we used xgboost in order to find out the importance of each feature.

**Table 1:** List of discrete features extracted from enterprise data warehouse.

Demographics	Age, gender, race, ethnicity, marital status, and insurance status
Risk factors	Hypertension, diabetes mellitus, atrial fibrillation, prior stroke, coronary artery disease, congestive heart failure, valvular heart disease, coronary artery bypass graft/stent, end-stage renal disease, hypothyroidism, dementia, cancer, chronic lung disease, and smoking status
Index stroke encounter characteristics	Primary stroke type, initial NIHSS score, initial GCS score, in-hospital pneumonia, medications (e.g., anticoagulants) at discharge, percutaneous endoscopic gastrostomy, mechanical ventilation, intensive care unit stay, and discharge destination
Other baseline factors	Miles from residence to hospital, frequency of hospital admissions in preceding year, and frequency of stroke admissions in preceding year


Next, we constructed three different types of NLP features from the unstructured clinical notes. To do that, we first pre-processed the notes to remove language abnormalities and make it usable for feature extraction. Specifically, we lowercased the text, removed punctuations, and stop words and non-alphanumeric words. We aggregated all the reports for each patient and then created a large corpus of all the aggregated reports from all the patients. We then created a token dictionary of all the unique important terms from the corpus. We experimented with unigrams, bigrams, trigrams, and noun phrases; however, we found the combination of unigrams and bigrams to work best. An *n*-gram is a set of occurring words within a given window (for example, *n* = 1 it is unigram, *n* = 2 it is bigram, *n* = 3 it is trigram, and so on).

For our first set of NLP features, using the token dictionary, we transformed the corpus to a patient-token matrix in which each token (unigram or bigram) is represented by term-frequency-inverse document frequency (tf-idf). Next, we used logistic regression with “l1” penalty (LASSO) to reduce the large dimensionality of features (13). The LASSO method puts a constraint on the sum of the parameter coefficient and applies shrinking (regularization) to penalize the coefficient of non-essential features to zero. We filtered all the non-zero coefficient features and used them as our second set of features.

For second set of features, on the patient-token matrix, we applied principal component analysis (PCA) (14) and constructed a graph of the variance by cumulative number of principal components. This graph provided us with the most effective number of principal components that explained the most variance in the data set. We then selected these principal components to form our third set of features.

For final set of features, we ran word2vec (15) on the text corpus to learn word vectors for each token in our dictionary. We used genism (16) package and continuous bag of words approach with standard parameters for running word2vec algorithm. Next, to construct a patient vector, we summed all the individual token vectors for each token present in each patient's report. Doing this, each patient is then represented by a single vector, which formed our fourth and final set of features.

### Definition of Outcomes

Readmission was defined as any unplanned inpatient hospitalization for any cause after index stroke hospitalization discharge. We excluded planned or scheduled readmissions, emergency department visits without admission, and observation visits. Using the date of index stroke hospital discharge and date of readmission, we identified unplanned readmissions occurring within 30 days of hospital discharge.

### Predictive Models

We developed models to predict two separate outcomes: (1) 30-day all-cause readmission and (2) 30-day stroke readmission. For each of these outcomes, we trained different predictive models and compared them with each other. In addition, we also used different types of features for each of predictive models as discussed above. Thus, our study not only evaluates the performance of different predictive algorithms but also the added value of different types of features. We trained a number of different base predictive models as well as several hierarchical predictive models to enhance predictive performance. The base models included logistic regression (17), naïve Bayes (18), support vector machines (19), random forests (18), gradient boosting machines (20), and finally extreme gradient boosting (XGBoost) (21). We trained each of these models for each of the feature types and compared the performance across multiple models.

**Figure 1:**
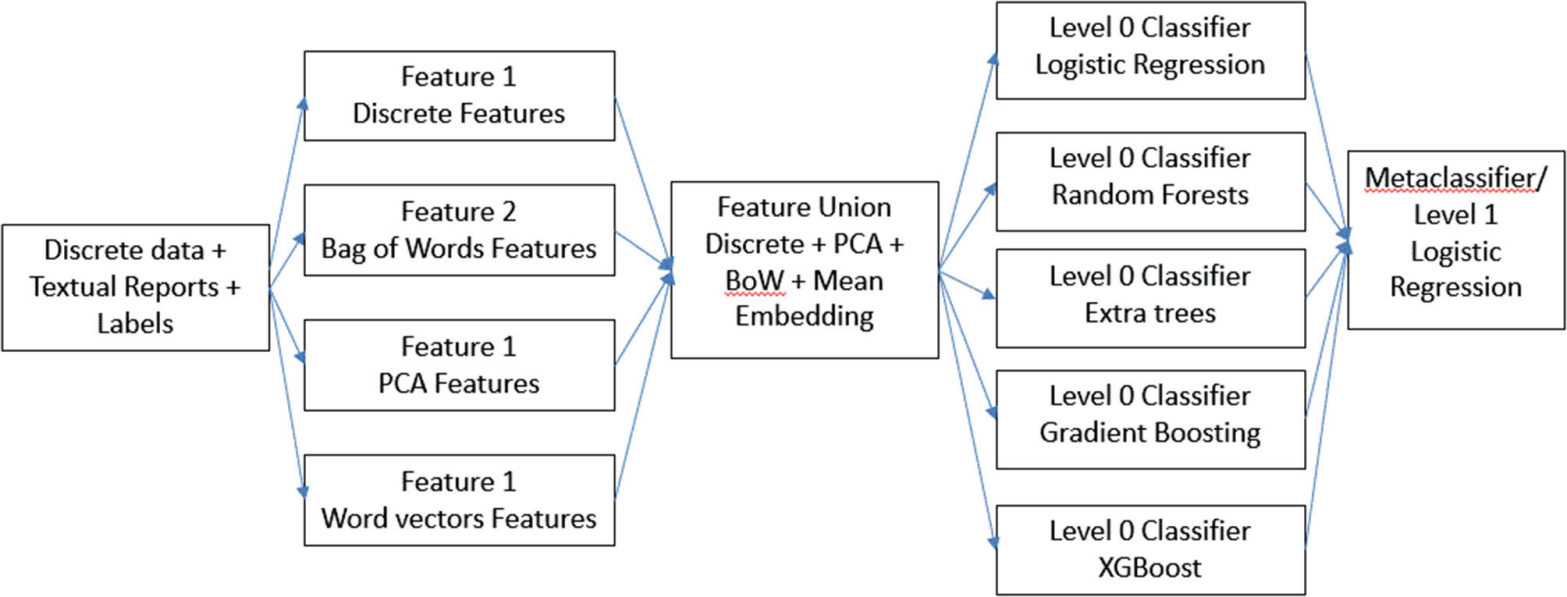
Description of feature ensemble method.

For our first hierarchical model (Figure 1), we combined all the features in the dataset to form a “super” feature set and then trained each of the base models on top of it. In addition, we combined the results from each of these base models and using those as features, we trained another meta-classifier model. We experimented with logistic regression as well as XGBoost for meta-classifier, but we found logistic regression to perform better. We designated this model a feature ensemble model.

**Figure 2:**
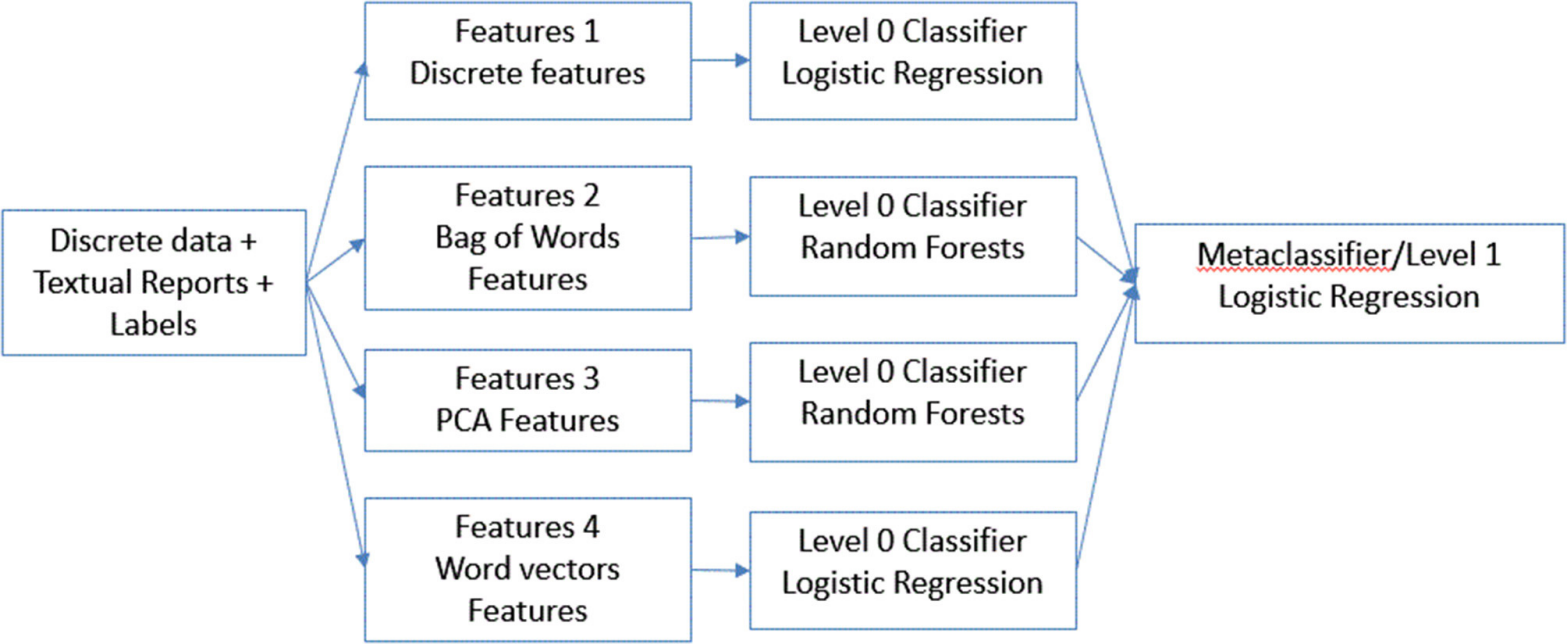
Description of classifier ensemble method.

Next, for our final model (Figure 2), instead of combining all the features, we concatenated results from the best performing model on individual features. We used the predictions from each of these models as features to train a meta-classifier. This technique is known as stacking (22) wherein outputs from base predictive models are combined to form a feature set which is then used to train another level 2 classifier. We designated this method a classifier ensemble model.

### Validation and Evaluation

To avoid over-fitting, we performed five-fold cross-validation (23). Cross-validation, also called rotation estimation, is a technique to evaluate predictive models by partitioning the original sample into a training set to train the model and a validation set to evaluate it. In *k*-fold cross-validation, the original sample is randomly partitioned into *k* equal size subsamples. Of the *k* subsamples, a single subsample is retained as the validation data for testing the model, and the remaining *k*-1 subsamples are used as training data. The cross-validation process is then repeated *k* times (the folds), with each of the *k* subsamples used exactly once as the validation data. The results from the folds can then be averaged (or otherwise combined) to produce a single estimation. We also performed hyper-parameter tuning for our base model within each fold using “hyperopt” python package (24).

In order to test true generalizability of our results, we obtained another dataset spanning from January 1, 2016 to December 31, 2016. We pre-processed it the same way as we did for training data we used for 5-fold cross validation. Next, we trained the best performing models for both outcomes on all the training data and performed the trained model in the test dataset to generate final predictions. We also bootstrapped the test dataset over 50 iterations to generate confidence intervals.

To evaluate the performance of each model, we estimated area under the curve or AUCs from receiver operating characteristic curve analysis. We also compared the best performing model with the baseline logistic regression model of discrete variables alone. *p*-values < 0.05 were considered significant in all analyses.

### Interpretability of NLP Features

To evaluate which NLP-based features were helpful in the prediction model, we ranked the bag of words features according to the feature importance given by the model.

### Standard Protocol Approvals, Registrations, and Patient Consents

This study was approved by the Institutional Review Board of Northwestern University. Informed consent was waived for this retrospective data analysis.

### Data Availability

All data not presented in this paper will be made available in a trusted data repository or shared at the request of other investigators for purposes of replicating procedures and results.

## Results

After pre-processing and combining various data files, we had 2,305 patients for training and 550 patients for testing. The mean age for training cohort and testing cohort was 64.4 and 64.8 years, respectively. The training and testing datasets were similar except the testing set contained more Hispanic, government-insured, married, hypertensive, cardiac disease, and intracerebral hemorrhage patients with more ICU days; the testing set also contained more patients who required acute inpatient rehabilitation at discharge (Table 2).

**Table 2:** Baseline characteristics of the training cohort (*n* = 2,305) and testing cohort (*n* = 550).

**Characteristic**	**Training cohort**	**Testing cohort**	***P*-value**
Mean age in years (SD)	64.4 (16.4)	64.8 (15.1)	0.90
Male sex [*n* (%)]	1,156 (50.2)	297 (54)	0.11
**Race [*****n*** **(%)]**			
White	1,156 (50.2)	284 (51.6)	0.09
Black	613 (26.6)	138 (25)	
Asian	78 (3.4)	13 (2.4)	
American Indian or Alaskan Native	4 (0.2)	4 (0.7)	
Native Hawaiian/Pacific Islander	4 (0.2)	3 (0.5)	
Declined, missing, or unknown	233 (10.1)	63 (11.4)	
Other	217 (9.41)	45 (8.1)	
Hispanic [*n* (%)]	164 (7.1)	63 (11.4)	<0.01
**Marital status [*****n*** **(%)]**			
Married	1,001 (43.4)	265 (48.1)	0.02
Widowed	253 (11.0)	45 (8.1)	
Single	759 (32.9)	157 (28.5)	
Divorced	142 (6.2)	33 (6)	
Separated	8 (0.3)	1 (0.2)	
Unknown, other, or missing	142 (6.2)	49 (8.9)	
**Insurance status [*****n*** **(%)]**			
Private	833 (36.1)	173 (31.5)	<0.01
Medicare	1,060 (46.0)	278 (50.5)	
Medicaid	182 (7.9)	63 (11.5)	
Other or self-pay	230 (10.0)	36 (6.5)	
**Primary index stroke diagnosis [*****n*** **(%)]**			
Ischemic stroke	1,825 (79.1)	416 (75.6)	<0.01
Intracerebral hemorrhage	257 (11.1)	94 (17)	
Subarachnoid hemorrhage	223 (9.7)	40 (7.3)	
Hypertension [*n* (%)]	1,853 (78.8)	466 (84.7)	0.01
Diabetes mellitus [*n* (%)]	629 (27.3)	179 (32.6)	0.13
Atrial fibrillation [*n* (%)]	430 (18.7)	111 (20.2)	0.42
Coronary artery disease [*n* (%)]	189 (8.2)	30 (5.5)	0.03
Congestive heart failure [*n* (%)]	232 (10.1)	67 (12.2)	0.15
Valvular heart disease [*n* (%)]	42 (1.8)	36 (6.5)	<0.01
Prior stroke [*n* (%)]	218 (9.5)	57 (10.3)	0.57
Chronic lung disease [*n* (%)]	236 (10.2)	48 (8.7)	0.29
Dementia [*n* (%)]	149 (6.5)	37 (6.7)	0.87
Cancer [*n* (%)]	180 (7.8)	45 (8.2)	0.75
End-stage renal disease [*n* (%)]	39 (1.7)	13 (2.3)	0.34
Hypothyroidism [*n* (%)]	270 (11.7)	56 (10.2)	0.32
**Smoking [*****n*** **(%)]**			
Current	363 (15.7)	76 (13.8)	0.03
Former	595 (25.8)	115 (20.9)	
Non-smoker	1,224 (53.1)	328 (59.6)	
Missing or other	123 (5.3)	31 (5.6)	
Any prior hospitalization [*n* (%)]	1,428 (61.0)	324 (58.9)	0.37
Median initial NIHSS score (IQR)	2 (0–6)	2 (0–6)	0.09
Median initial GCS (IQR)	15 (14–15)	15 (14–15)	0.10
Missing [*n* (%)]	83 (3.6)	22 (4)	0.65
Intensive care unit stay [*n* (%)]	1,166 (50.6)	306 (55.64)	0.04
Inhospital pneumonia [*n* (%)]	108 (4.7)	24 (4.4)	0.76
Mechanical ventilation [*n* (%)]	226 (9.8)	49 (8.9)	0.52
Gastrostomy [*n* (%)]	153 (6.6)	35 (6.3)	0.80
**Discharge destination [*****n*** **(%)]**			
Home	1,659 (72.0)	350 (63.6)	<0.01
Acute inpatient rehabilitation	429 (18.6)	148 (26.9)	
Skilled nursing facility or long-term facility	153 (6.6)	33 (6)	
Other hospital or against medical advice	64 (2.8)	19 (3.45)	
Any unplanned readmission within 30 days [*n* (%)]	337 (14.6)	62 (11.5)	0.04
Stroke readmission within 30 days [*n* (%)]	124 (5.4)	24 (4.5)	0.33

In training cohort, there were 337 patients (14.6%) with all-cause readmission within 30 days and 124 patients (5.4%) with stroke readmission within 30 days. In testing cohort, there were 62 patients (11.3%) with all-cause readmission within 30 days and 24 patients (4.4%) with stroke readmission within 30 days. We collected ~28,500 different patient reports for the training data set and 6,606 reports for the test dataset. We extracted 35 discrete features, 250 principal components features, 400 word-vector features, and 200 bag of words features for all patients in both cohorts.

**Figure 3:**
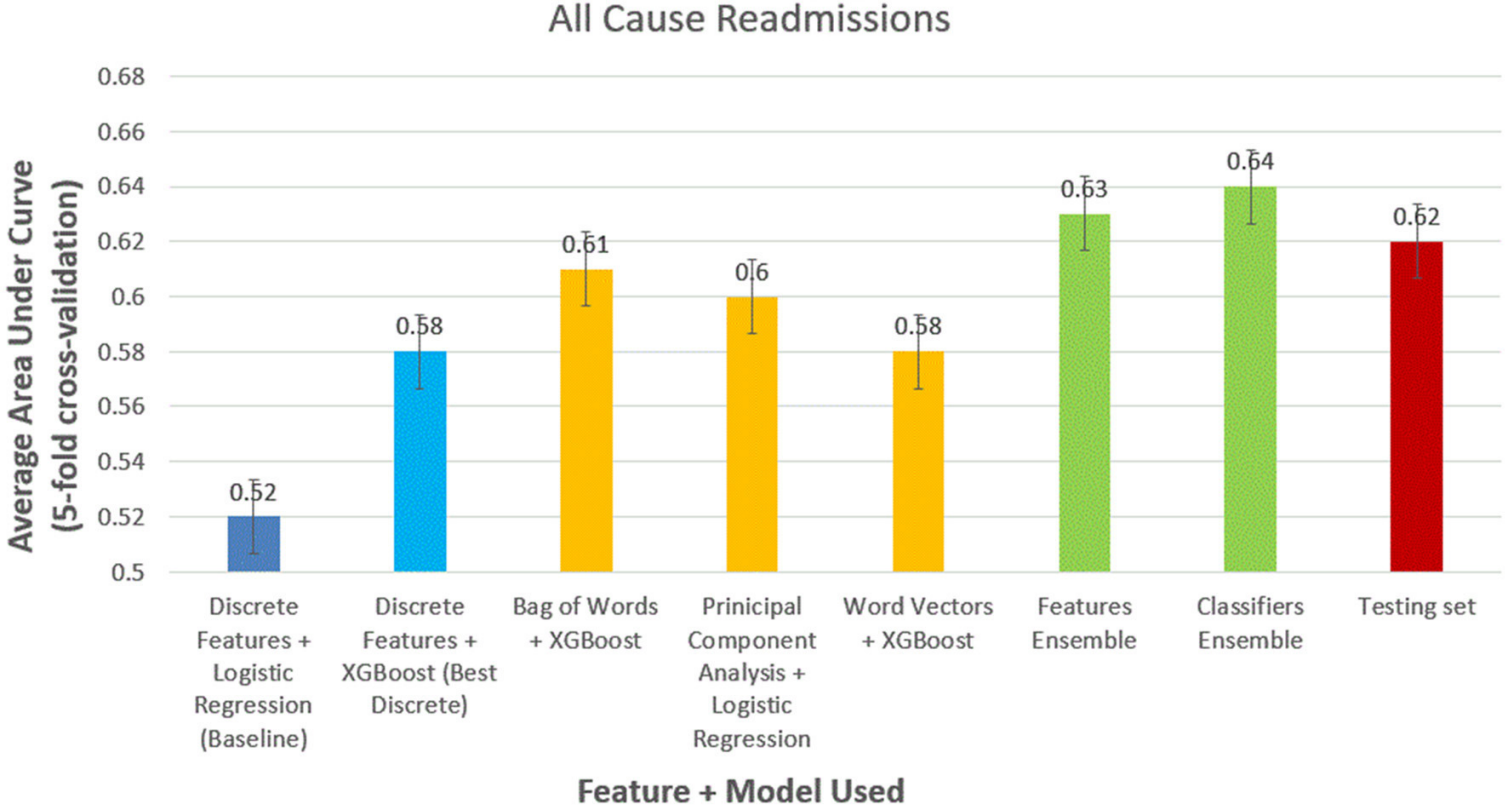
Comparison of models to predict 30-day all-cause readmissions.

For all-cause readmission (Figure 3), a model using logistic regression using discrete features had AUC of 0.58 (95% CI, 0.57–0.59). In comparison, XGBoost outperformed logistic regression using the same discrete features with an AUC of 0.62 (95% CI, 0.61–0.63). Using NLP-based features, we obtained similar results with XGBoost performing best with bag of words features (AUC, 0.61; 95% CI, 0.60–0.62), logistic regression performing best with PCA features scoring (AUC, 0.61; 95% CI, 0.59–0.62), and XGBoost performing best with word-vector-based features (AUC, 0.60; 95% CI, 0.59–0.61). Ensemble model performed best with feature ensemble method (AUC, 0.64; 95% CI, 0.62–0.66) and classifier ensemble method (AUC, 0.65; 95% CI, 0.62–0.66). We performed the trained classifier ensemble model in the test dataset with bootstrapping over 50 iterations, which resulted in an AUC of 0.64 (95% CI, 0.63–0.65).

**Figure 4:**
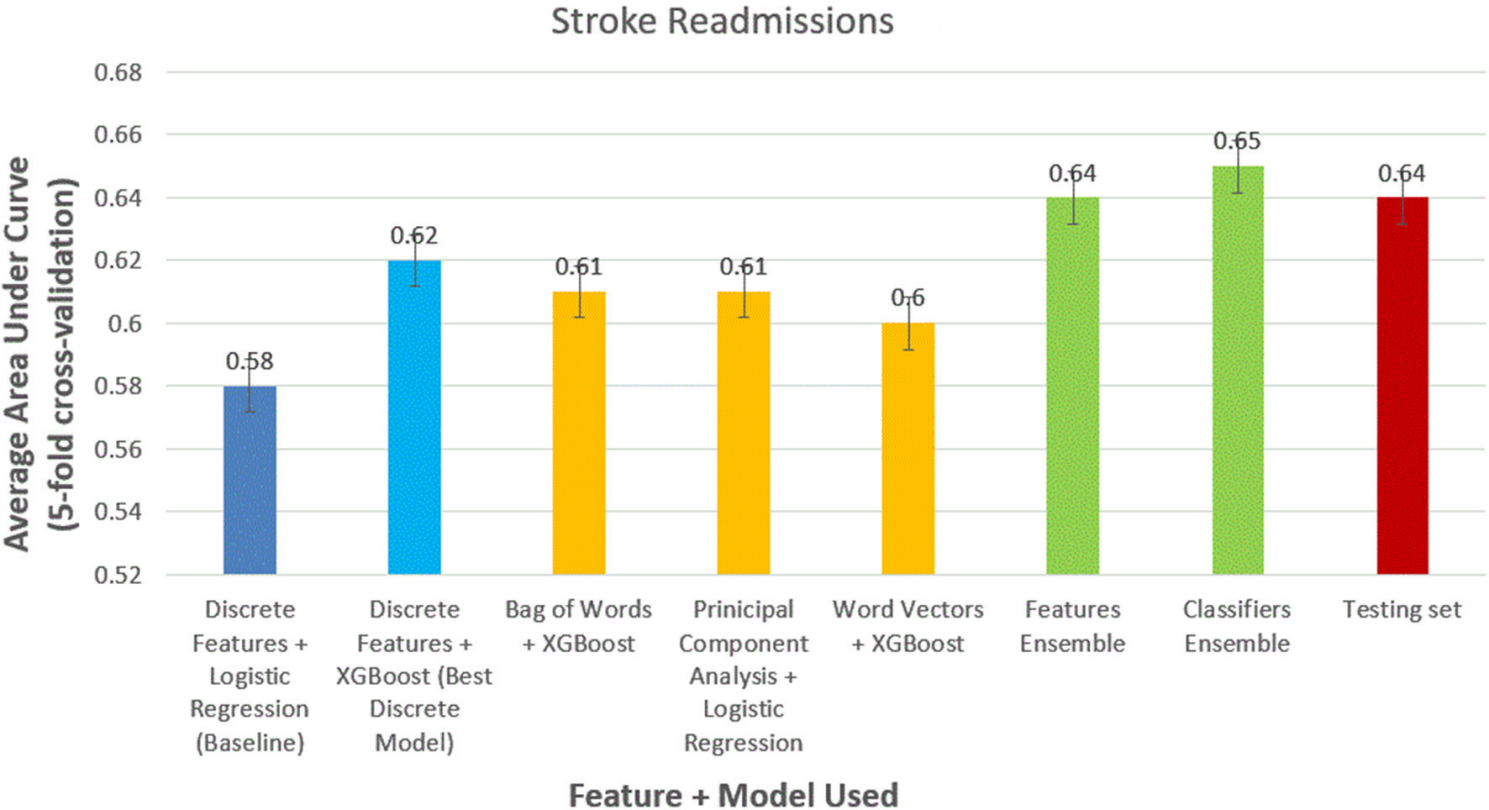
Comparison of models to predict 30-day stroke readmissions.

We obtained similar results for 30-day stroke readmissions (Figure 4). Logistic regression with discrete features formed modest baseline with AUC of 0.52 (95% CI, 0.51–0.53). XGBoost outperformed logistic regression using discrete features alone with AUC of 0.58 (95% CI, 0.56–0.59). The models using the best NLP-based features produced AUCs of 0.61 (95% CI, 0.59–0.63), 0.60 (95% CI, 0.59–0.62), and 0.58 (95% CI, 0.57–0.59) for bag of words features, PCA features, and word-vector features, respectively. Ensemble methods were again the best performing models with AUCs of 0.63 (95% CI, 0.6–0.65) and 0.64 (95% CI, 0.62–0.66) for feature ensemble model and classifier ensemble models, respectively. Performed on the test set, we obtained an AUC of 0.62 (95% CI, 0.61–0.63) using classifier ensemble.

Some of the NLP features that were ranked higher in importance by the model were as follows: “stenosis,” “encephalomalacia,” “craniectomy,” “encephalomalacia,” “mild calcified atherosclerotic,” “hypoattenuation white matter,” and “chiari ii malformation.”

## Discussion

Given the burden of readmission on the patient and the healthcare system, improving prediction of readmissions with a goal of preventing them is of major importance. A prior study estimated that the cost to Medicare of unplanned rehospitalizations in 2004 was $17.4 billion (25). Readmission to the hospital within 30 days after stroke is also associated with 1-year mortality and serves as a quality metric across specialties under the guidance of the Affordable Care Act (3, 26).

Currently, clinician judgment and simple mathematical models are able to only modestly predict readmission after stroke. In our study, the baseline model that used logistic regression and discrete variables resulted in poor discrimination of 30-day readmission, a result that is consistent with prior studies (5, 7, 8). While NLP-enhanced ML models advance conventional approaches, further improvement is necessary before these predictive models can be implemented in practice given the weak discrimination. Our finding is similar to another study using machine learning in readmission after heart failure (11).

Given the challenges in accurate prediction of 30-day readmission even using modern machine learning approaches, grading and penalizing hospitals on this metric may not be justifiable. Indeed, hospitals may be forced to “game” the system by increasing observation status visits and avoid penalties at the cost of increasing mortality as a recent study in heart failure patients found (27). Therefore, the penalties facing hospitals seem misguided until such a time when readmission prediction is more robust.

Machine learning is able to weigh the interactions between complex variables in additive analysis to produce better prediction models. In addition, the use of NLP in medicine may be revolutionary. Untangling the complex data within clinical notes and other non-discrete and unstructured data could be valuable in tackling a myriad of research questions. Our advanced models could further ongoing machine learning efforts across specialties to better identify patients for clinical trials, radiologic findings in neurologic emergencies, dermatologic-related malignancies, automatic infectious disease prediction in the emergency room, and outcomes in psychiatric admissions (28–32).

The strengths of our study include a five-fold cross-validation technique to avoid overfitting. The internal validity of our results was further tested by obtaining a second dataset not used in the derivation and validation steps. We also bootstrapped the test dataset over 50 iterations to generate confidence intervals. Our study, however, has limitations. ML algorithms are also limited by the data that are fed into them such that data that are not commonly reflected in the EHR, such as psychosocial factors, post-discharge care coordination, detailed social support post-hospitalization, and post-stroke rehabilitation care are not accounted for in our study. Prior studies suggest including post-acute care data improve prediction of readmission (5, 33). Healthcare systems across the country are heterogeneous, and the variables we used may be non-uniformly available at other hospitals. External validation of our results is necessary. An additional limitation of a single-center cohort is the potential for incomplete follow-up (e.g., care fragmentation leading to admission at another hospital in the region) resulting in an underestimation of readmission rates. However, a recent Chicago multihospital study noted a low rate of care fragmentation (34). There are several differences between the two datasets: the training dataset as it was later chronologically noted changes in the health system and stroke program. These differences may result in error in trained model validation. However, it does provide some measure of external validation as the model performed well. Nevertheless, formal external validation of the model is recommended. In addition, these algorithms require large volume, structured pools of data. Approximately 80% of EHR data is composed of provider notes. Our use of NLP provided a tool for deconstructing these language blocks; however, sufficient time is required to design and train these programs (9). Lastly, these programs lack the clinical insight that is essential for unsupervised implementation, and with any “black box” program, results must be interpreted cautiously (11).

## Summary

In summary, we demonstrated a modest added utility of NLP-enhanced ML algorithms to improve prediction of 30-day readmission after stroke hospitalization compared with conventional statistical approaches using discrete predictors alone. While these results are encouraging, further work is required. Given the challenges in predicting readmission after stroke even using the most advanced techniques, the current penalties applied to hospitals for unplanned readmissions should be reevaluated.

## Data Availability Statement

The datasets presented in this article are not readily available because the code and data contains Protected Health Information (PHI). Requests to access the datasets should be directed to the corresponding author.

## Author Contributions

Statistical analysis was done by RG. All authors contributed to the article and approved the submitted version.

## Conflict of Interest

The authors declare that the research was conducted in the absence of any commercial or financial relationships that could be construed as a potential conflict of interest.
